# Nicotinic Acid Increases Adiponectin Secretion from Differentiated Bovine Preadipocytes through G-Protein Coupled Receptor Signaling

**DOI:** 10.3390/ijms151121401

**Published:** 2014-11-18

**Authors:** Christina Kopp, Afshin Hosseini, Shiva P. Singh, Petra Regenhard, Hamed Khalilvandi-Behroozyar, Helga Sauerwein, Manfred Mielenz

**Affiliations:** Institute of Animal Science, Physiology & Hygiene Unit, University of Bonn, 53115 Bonn, Germany; E-Mails: christina.kopp@uni-bonn.de (C.K.); afshin.hosseini@gmail.com (A.H.); spsinghmail1@gmail.com (S.P.S.); Petra.Regenhard@lohmann.de (P.R.); khalilvandi@ut.ac.ir (H.K.-B.); sauerwein@uni-bonn.de (H.S.)

**Keywords:** adiponectin, 5'-adenosine monophosphate-activated protein kinase, bovine adipocytes, G-protein coupled receptor 109A, nicotinic acid

## Abstract

The transition period in dairy cows (3 weeks prepartum until 3 weeks postpartum) is associated with substantial mobilization of energy stores, which is often associated with metabolic diseases. Nicotinic acid (NA) is an antilipolytic and lipid-lowering compound used to treat dyslipidaemia in humans, and it also reduces non-esterified fatty acids in cattle. In mice the G-protein coupled receptor 109A (GPR109A) ligand NA positively affects the secretion of adiponectin, an important modulator of glucose and fat metabolism. In cattle, the corresponding data linking NA to adiponectin are missing. Our objective was to examine the effects of NA on adiponectin and AMPK protein abundance and the expression of mRNAs of related genes such as chemerin, an adipokine that enhances adiponectin secretion *in vitro*. Differentiated bovine adipocytes were incubated with pertussis toxin (PTX) to verify the involvement of GPR signaling, and treated with 10 or 15 µM NA for 12 or 24 h. NA increased adiponectin concentrations (*p* ≤ 0.001) and the mRNA abundances of GPR109A (*p* ≤ 0.05) and chemerin (*p* ≤ 0.01). Pre-incubation with PTX reduced the adiponectin response to NA (*p* ≤ 0.001). The NA-stimulated secretion of adiponectin and the mRNA expression of chemerin in the bovine adipocytes were suggestive of GPR signaling-dependent improved insulin sensitivity and/or adipocyte metabolism in dairy cows.

## 1. Introduction

The peripartal period is associated with manifold endocrine and metabolic changes to adapt the cow for parturition and lactogenesis. In dairy cows the transition from late pregnancy to early lactation, defined as 3 weeks prepartum until 3 weeks postpartum, is attributed to increased energy demand due to fetal growth and lactogenesis; the energy requirement exceeds dietary energy intake [1]. Energy stores are mobilized, lipogenesis is reduced and lipolysis is increased substantially [2]. High non-esterified fatty acids (NEFA) concentrations are considered as one major risk factor for metabolic diseases, such as fatty liver and ketosis [3,4] in dairy cows during the transition period [5]. The understanding of the regulation of lipolysis and adipocyte metabolism is therefore fundamental to cope with production diseases around parturition.

Nicotinic acid (NA), also known as Niacin; has been recognized as a high-affinity ligand for the G_i_/G_o_-protein-coupled receptor 109A (GPR109A) in non-ruminants [6,7,8]. Nicotinic acid reduces triglycerides and low-density lipoprotein cholesterol and increases high-density lipoprotein cholesterol [9]. The seven transmembrane GPR109A, also known as hydroxycarboxylic acid receptor 2 (HCA_2_), is activated by its endogenous ligand beta-hydroxybutyrate [10,11]. GPR109A is expressed in activated macrophages and in adipocytes [8,12] and has also been detected in bovine tissues [13]. Decreases in NEFA and beta-hydroxybutyric acid concentrations in dairy cows have been shown following treatment with NA [14,15,16,17]. The secretion of the adipokine adiponectin is stimulated by NA in rodents [18,19]. Several studies have demonstrated NA-induced increases in the expression and secretion of the adiponectin protein [20,21]. The secretion of adiponectin is inhibited by pertussis toxin (PTX), a G_i_/G_o_-protein-un-coupling compound, in 3T3-L1 adipocytes *in vitro*, and similar results have been obtained in GPR109A knockout mice; these findings support the involvement of GPR-signaling in this pathway [18]. Adiponectin is primarily expressed in adipocytes and modulates the glucose and lipid metabolism of insulin-sensitive tissues [22]. Adiponectin exerts its effects via binding to its receptors AdipoR1/R2 and the subsequent activations of the peroxisome proliferator-activated receptor α (PPARα) and 5'-adenosine monophosphate-activated protein kinase (AMPK) [23]. AMPK is a heterotrimeric kinase complex that consists of a catalytic α subunit and regulatory β and γ subunits [24]. This kinase is activated via the phosphorylation of threonine 172 in the α-subunit (pAMPK) [25]. Upon activation, AMPK acts as an intra-cellular energy sensor that turns on catabolic pathways (e.g., fatty-acid oxidation and glycolysis pathways) and inhibits anabolic processes, such as the syntheses of cholesterol, glycogen, and protein. Chemerin is synthesized as a proprotein and is a chemoattractant agent that is highly expressed in AT and the liver [26]. Chemerin modulates the innate immune system in both directions by acting as a pro- and anti-inflammatory protein [27,28]. Chemerin has also been identified as an adipokine that regulates adipogenesis and adipocyte metabolism [26]. In 3T3-L1 adipocytes, chemerin has been reported to enhance insulin-stimulated glucose uptake and adiponectin secretion [26,29] and thereby to positively regulate insulin sensitivity. Song *et al.* [30] recently cloned bovine chemerin and characterized its expression. These authors observed an increase in expression throughout the differentiation of bovine adipocytes *in vitro*. Data regarding the effects of NA treatment on chemerin expression in bovine adipocytes is lacking.

In the present study, we hypothesized that NA affects adiponectin secretion and AMPK activation in bovine adipocytes. Furthermore, we analyzed the mRNA abundances of genes relating to NA and adiponectin signaling (e.g., GPR109A, AdipoR1/R2, and chemerin). For this purpose, a primary cell culture system consisting of differentiated bovine adipocytes was established. To investigate the importance of NA on GPR signaling in cows, the adipocytes were pre-incubated with PTX.

## 2. Results

### 2.1. Effect of NA on Adiponectin Secretion

**Figure 1:**
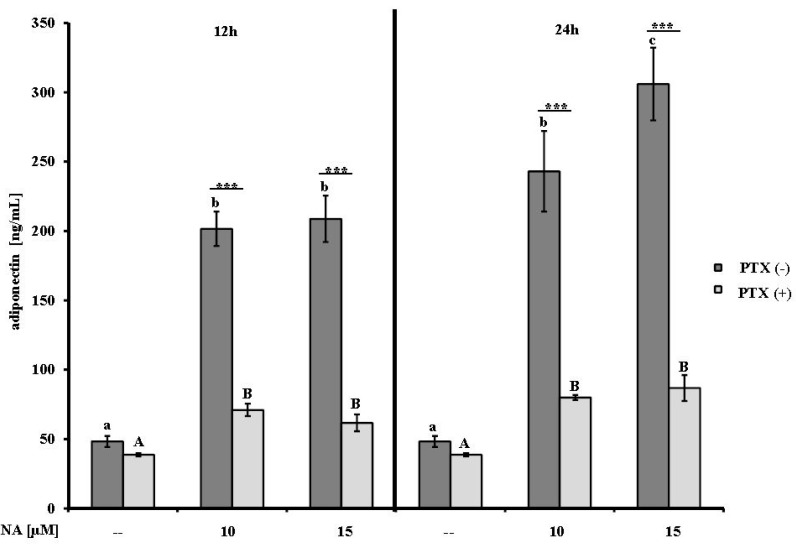
The effects of nicotinic acid (NA) on adiponectin concentrations (means ± SEM) in cell culture supernatants of differentiated bovine adipocytes (*n* = 5). After 4 h of serum starvation, the adipocytes were pre-incubated with pertussis toxin (PTX (+)) or without (PTX (−)) (100 ng/mL) for 16 h and then treated for 12 or 24 h with 10 or 15 µM NA or PBS (vehicle control). The different lower case letters designate significant differences (*p* ≤ 0.005) between the NA treatments and the controls for the PTX (−) cells; the different upper case letters designate significant differences (*p* ≤ 0.001) between the NA treatments and the controls for the PTX (+) cells. Significant differences (*** *p* ≤ 0.001) due to PTX (+) or PTX (−) pre-incubation for each NA treatment group are indicated with asterisks.

To test whether NA affects the secretion of adiponectin, the differentiated bovine adipocytes were stimulated with two different concentrations of NA (10 and 15 µM) for 12 or 24 h. Compared to the NA-free controls, the adiponectin concentrations in the cell culture supernatants were 4-fold increased following stimulation with 10 or 15 µM NA for 12 h (*p* ≤ 0.001) and approximately 5- and 6-fold increased following 24 h treatments with 10 and 15 µM NA, respectively (*p* ≤ 0.001) (Figure 1). To assess whether the effects of NA were mediated by GPR signaling, the experiments were performed following pre-incubation with PTX (100 ng/mL) for 16 h. For both NA doses and both durations of treatment, the pre-incubation with PTX decreased the adiponectin concentrations to values between 62 and 87 ng/mL; however, these concentrations were consistently higher than those observed in the relevant NA-free control (39 ng/mL; *p* ≤ 0.001). Comparisons of the PTX pre-incubation groups revealed that the adiponectin concentrations were approximately 3 times lower following PTX pre-incubation in the 10 µM NA group at both durations (*p* ≤ 0.001). The adiponectin concentrations in the supernatants from the PTX (+) group were reduced by 3- and 3.5-fold (*p* ≤ 0.001) following treatment with 15 µM NA regardless of time when compared with the corresponding PTX (−) group (Figure 1).

### 2.2. Effect of NA on AMPK

**Figure 2:**
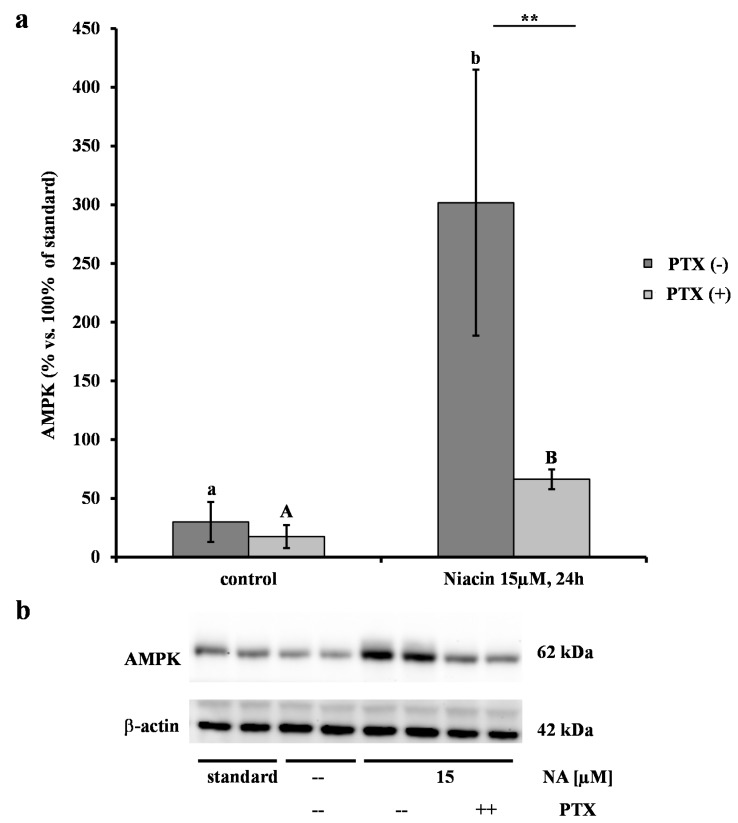
Effects of nicotinic acid (NA) on 5' AMP-activated protein kinase (AMPK) protein abundance (means ± SEM) in differentiated bovine adipocytes (*n* = 5). After 4 h of starvation, the adipocytes were pre-incubated with (PTX (+)) or without pertussis toxin (PTX (−)) (100 ng/mL) for 16 h and then treated for 24 h with 15 µM NA or PBS (control). (**a**) The different lower case letters designate significant differences (*p* ≤ 0.001) between the NA treatments and the controls for the PTX (−) cells. The different upper case letters designate significant differences (*p* ≤ 0.001) between the NA treatments and the controls for the PTX (+) cells. Significant differences (** *p* ≤ 0.01) due to (+) or (−) PTX pre-incubation are designated with asterisks; (**b**) Representative Western blot results. After gel electrophoreses, the membranes were incubated with specific antibodies against AMPK or β-actin as a loading control. The obtained optical densities for AMPK were matched against a standard pool sample and are expressed as % relative to the standard.

The phosphorylated form of AMPK remained undetected, therefore the obtained optical densities of AMPK were matched against the reference standard. Treatment with 15 µM NA for 24 h increased the AMPK/standard ratio 10-fold compared to the control (*p* ≤ 0.001) (Figure 2a). Pre-incubation with PTX drastically limited this increase by 50%, but the values remained 5-fold higher than those of the respective controls (*p* ≤ 0.001). A representative Western blot result is shown in Figure 2b.

### 2.3. Effects of NA on the Abundances of AdipoR1/2, FABP4, and GPR109A mRNAs

**Figure 3:**
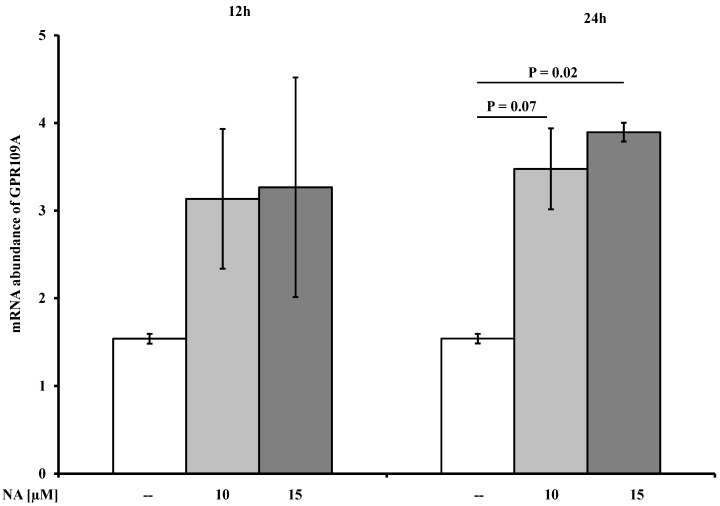
The effects of nicotinic acid (NA) on the mRNA abundance of G-protein coupled receptor 109A (GPR109A) in differentiated bovine adipocytes cells (*n* = 10). After 4 h of starvation, the adipocytes were pre-incubated with (PTX (+)) or without pertussis toxin (PTX (−)) (100 ng/mL) for 16 h and then treated for 12 or 24 h with 10 or 15 µM NA or PBS (control). Due to the absence of differences, the PTX (+) and PTX (−) groups were merged for the analyses of the mRNA abundances. Significant differences (*p* ≤ 0.05) and trends (*p* ≤ 0.1) are designated with the corresponding *p*-values.

Pre-incubation with PTX had no effect on the mRNA abundances of AdipoR1/2, FABP4, or GPR109A. Therefore, the PTX (+) and PTX (−) groups were merged for further analyses. Compared to the controls, incubation for 24 h with 15 µM NA increased the mRNA abundance of GPR109A (*p* ≤ 0.05), and a trend (*p* = 0.07) toward an increase was observed following treatment with 10 µM NA after 24 h (Figure 3). In contrast, treatment with NA had no effect on the mRNA abundances of AdipoR1/R2 or FABP4 (Table 1).

**Table 1:** Niacin effects on the mRNA abundance of selected genes.

Treatment/Genes	12 h					24 h				
NA ^a^ [μM]	-	10	*p*	15	*p*	-	10	*p*	15	*p*
AdipoR1 ^b^	1.1 ± 0.4	1.1 ± 0.5	n.s.	1.1 ± 0.4	n.s.	1 ± 0.3	1.1 ± 0.3	n.s.	1.4 ± 0.5	n.s.
AdipoR2 ^c^	1 ± 0.1	1 ± 0.2	n.s.	1 ± 0.1	n.s.	1 ± 0.2	1 ± 0.2	n.s.	1 ± 0.1	n.s.
GPR109A ^d^	1.2 ± 0.01	3.13 ± 0.7	n.s.	3.27 ± 1.25	n.s.	1.6 ± 0.05	3.48 ± 0.46	0.07	3.9 ± 0.1	0.02
FABP4 ^e^	1.2 ± 0.3	1.2 ± 0.6	n.s.	1.1 ± 0.6	n.s.	1.1 ± 0.5	0.6 ± 0.5	n.s.	0.8 ± 0.5	n.s.
Chemerin	0.71 ± 0.19	0.76 ± 0.16	n.s.	0.67 ± 0.38	n.s.	0.76 ± 0.46	2.89 ± 1.42	0.006	1.59 ± 1.18	n.s.

The given values are means ± SEM. Significant differences (*p* ≤ 0.05 and *p* ≤ 0.1 as a trend) between control and NA treatment are depicted by bold values. Pre-incubation with pertussis toxin (PTX) had no effect of the analyzed mRNAs of AdipoR1/2, FABP4 or GPR109A. Therefore, the PTX (+) and PTX (−) groups were merged for further analyses. The mRNA abundance of chemerin is presenting only data of the PTX (−) group; ^a^ NA: Niacin; ^b^ AdipoR1: adiponectin receptor 1; ^c^ AdipoR2: adiponectin receptor 2; ^d^ GPR109A: G-protein coupled receptor 109A; ^e^ FABP4: fatty acid binding protein 4.

### 2.4. Effect of NA on the mRNA Abundance of Chemerin

The mRNA abundance of chemerin was increased by 3.3-fold (*p* ≤ 0.01) compared to the controls following stimulation with 10 µM NA for 24 h. No effects were observed following the 12 h treatment or the treatments with 15 µM NA for either duration. Following pre-incubation with PTX, no differences between any treatment group and the NA-free controls were observed (Table 1).

## 3. Discussion

During the transition period the energy required exceeds the dietary energy intake, and this time period is linked with the health status [1] and thus with the profitability of the dairy cow. Due to this negative energy balance, lipogenesis is reduced and lipolysis is substantially increased [2]. Excess non-esterified fatty acids (NEFA) accumulate, alter liver function and increase the incidence of metabolic diseases, such as fatty liver and ketosis [3,4]. In dairy cows, the NEFA- and beta-hydroxybutyric acid-lowering effects of NA were first shown many years ago [14,15]. Recently, in dairy cows, NA and beta-hydroxybutyric acid were demonstrated to decrease isoproterenol-stimulated lipolysis *in vitro* by reducing the phosphorylation of hormone-sensitive lipase, which confirmed the presence of a GPR109A-mediated anti-lipolytic pathway in dairy cows [31]. In addition to the effects on lipolysis, NA has been linked to elevated mRNA expression and protein secretion of adiponectin in murine 3T3-L1 cells [20] and humans [21], respectively. Adiponectin is known to improve insulin sensitivity and lipid metabolism [32], but information about the effects of NA on adiponectin in cattle is lacking.

With our *in vitro* model, we were able to demonstrate effects of NA on differentiated primary bovine adipocytes *in vitro.* Our results might link the effects of NA to insulin sensitivity at the level of the adipocyte in bovines in a process that involves adiponectin secretion and AMPK protein expression. We observed an increase in the adiponectin secretion, in differentiated bovine adipocytes *in vitro*, following two different NA treatments after 12 h and another enhancement after 24 h of incubation. These observations are in line with those from a study by Plaisance *et al.* [18] in which the stimulation of isolated primary rat adipocytes with NA increased adiponectin secretion. Adiponectin exerts its effects via binding to its AdipoR1/R2 receptors and activating PPARα, and AMPK [23]. Yamauchi *et al.* [25] reported increased phosphorylation of AMPK following stimulation with adiponectin in myocytes and hepatocytes that resulted in the stimulation of glucose uptake and fatty-acid oxidation. Recently, adiponectin has been shown to activate AMPK in bovine hepatocytes, and this activation results in increased lipid oxidation and reduced lipid synthesis [33]. To our knowledge, no detailed information is available regarding this issue in bovine adipocytes, but it has been demonstrated that the overexpression of adiponectin in 3T3-L1 adipocytes *in vitro* increases lipogenesis and lipid accumulation [34]. Data regarding the activation of AMPK in AT are contradictory because both lipogenic and lipolytic effects have been described [35,36]. In dairy cows, the phosphorylation of AMPK increases during the transition period. As discussed by Locher *et al.* [37], this observation might be associated with the antilipolytic function of AMPK in terms of the fine-tuning of NEFA release from triglycerides after parturition. However, the regulation of AMPK at the expression level was shown as response to physical activity or leptin administration [38,39,40,41]. In addition Martinez-Agustin *et al.* [42] demonstrated a direct correlation between AMPK protein abundance and adiponectin expression in human AT, supporting adiponectin as discrete activator of AMPK [43,44]. Similarly, treatment with 15 µM NA for 24 h increased the AMPK amount by up to 10-fold, which presumably resulted from an elevation in AMPK protein expression subsequent to the increased secretion of adiponectin following NA treatment. In addition to AMPK phosphorylation, the increase in AMPK abundance might be in line with the antilipolytic effects of NA that have been shown in dairy cows [14,15] and its effects on the fine-tuning of lipolysis [37]. The concentrations of NA used by Kenéz *et al.* [31] were discussed as reflective of physiological concentrations following NA feeding, and the concentrations we used were also within this range. In humans and rodents, NA exerts its lipid-lowering effects by binding to GPR109A [7]. Furthermore, mice that are deficient in PUMA-G (the mouse ortholog of GPR109A) exhibit no increase in serum adiponectin concentration following treatment with NA [7]. Titgemeyer *et al.* [13] identified GPR109A mRNA and protein in various bovine tissues. To test whether the modulating effects of NA on adiponectin and AMPK are mediated by GPR109A in cattle, we pre-incubated bovine adipocytes with PTX, a standard inhibitor of G_i_/G_o_-protein-coupling. Adiponectin secretion was significantly decreased after blocking G_i_/G_o_-protein signaling irrespective of the amount or duration of NA treatment. Adiponectin increases the abundance of AMPK [42] and might be therefore responsible for the decrease in the abundance of the AMPK amount in consequence of the treatment with PTX. However, adiponectin secretion was still significantly increased compared to the controls. This finding contrasts those of Plaisance *et al.* [18] in rat adipocytes; these authors observed no increase on adiponectin secretion following stimulation with NA after PTX pre-treatment. Therefore, we suggest that, in addition to GPR signaling-dependent pathways, GPR-signaling independent pathways are involved in the regulation of adiponectin secretion following stimulation with NA in bovines. In 3T3-L1 adipocytes, NA has been shown to increase PPARγ mRNA abundance [45,46]. An increase of PPARγ mRNA by NA was also observed in GPR109A knockout mice [19]. Kim and Choung [47] reported an up-regulation of the mRNA expression of PPARγ in AT following treatment with an extract of cinnamon bark, the main compounds of which are known to be ligands of GPR109A. The transcription factor PPARγ is a known stimulator of adiponectin expression [48] and a regulator of several genes that are involved in the control of insulin sensitivity [49]. In addition to an increase in PPARγ expression, an increase in adiponectin secretion has been observed following the administration of cinnamon extract [47]. These findings support the notion that this regulatory pathway is a possible NA-induced stimulator of adiponectin secretion independent of GPR109A signaling, and suggest PPARγ as an interesting target gene for further studies in the bovine. Corresponding to the results observed for adiponectin, the protein abundance of AMPK decreased following PTX incubation but remained 4-fold higher than the levels observed in the controls. These findings might be due to a subsequent effect of the PTX-independent increase in adiponectin concentration and its direct activation of AMPK [25]. The NAD/NADH redox potential as another metabolic sensor might be involved [50]. Nicotinic acid is a substrate for the synthesis of NAD^+^ after conversion to nicotinamide [51]. However, increasing NADH concentrations may down regulate AMPK activity [50]. The importance of this link on the abundance and activity of AMPK should be analysed in future experiments in the bovine.

In addition to the analysis of adiponectin and AMPK, the mRNA expression of related genes were quantified by PCR. FABP4 is widely known as a marker for mature adipocytes; it regulates the transport of NEFA and PPARγ agonists and also interacts with proteins linked to lipid metabolism and insulin sensitivity [52]. However, the mRNA abundance of FABP4 unexpectedly remained unchanged following treatment with NA. The effects of adiponectin are mediated through its receptors, although the mRNA abundance of AdipoR1/R2 did not change in our study. Therefore, neither NA nor adiponectin affected the receptor mRNA abundance in our *in vitro* model. Similarly, we have observed that adiponectin mRNA increased during the 13 d of adipocyte differentiation *in vitro* by 2500-fold in parallel with the relatively constant levels of the mRNAs of both adiponectin receptors [53]. Increased mRNA abundances of GPR109A were observed following 24 h of treatment with both NA concentrations. This effect was not blocked by PTX and was therefore not G_i_/G_o_-protein-coupling dependent. Another possibility could be signaling by PPARγ or stimulation of the NAD/NADH redox system by NA as discussed above. Correlation between PPARγ and GPR109A mRNA in epididymal white adipose tissue of mice has been shown *in vivo* [54], and the direct regulation of GPR109A by PPARγ in 3T3-L1 adipocytes was demonstrated by Jeninga *et al.* [55]. We recently have shown in dairy cows a high correlation between the mRNA abundance of PPARγ and GPR109A in subcutaneous adipose tissue (*p* = 0.782) but not in liver *in vivo* [56] which may support our speculation. All of the suggested mechanisms need to be checked in future experiments. The regulation of GPR109A mRNA by NA is in contrast with the results of the *in vivo* study by Titgemeyer *et al.* [13] who used Holstein steers and showed no alterations in GPR109A mRNA or protein expression following abomasal infusions of 16 g/d of NA in the AT or other tissues. The discrepancy between the results of our study that utilized differentiated adipocytes and those of Titgemeyer *et al.* [13] might be linked to the use of *in vitro vs. in vivo* models; this possibility should be clarified in further experiments. Chemerin is known to be involved in the control of immune responses via its action as a chemoattractant for antigen-presenting cells. Chemerin has anti-inflammatory and pro-inflammatory functions depending on the model studied [57]. The protein is highly expressed as prochemerin in the liver, AT and placenta [57] and was recently identified as an adipokine that regulates adipogenesis and adipocyte metabolism [58]. Furthermore, chemerin enhances insulin-stimulated glucose uptake, insulin signaling and adiponectin secretion and therefore improves insulin sensitivity in murine adipocytes [26,29]. Our study showed for the first time that treatment with 10 µM NA for 24 h increased the chemerin mRNA abundance 3-fold compared to the controls. We speculate that the increased chemerin mRNA might be indicative of enhanced adipocyte insulin sensitivity and/or improved adipocyte metabolism due to NA. Following pre-incubation with PTX, the chemerin mRNA abundance in the treatment group was similar to that of the controls, which confirms the hypothesized signaling pathway of NA through GPR signaling.

## 4. Experimental Section

### 4.1. Isolation of Bovine Preadipocytes

Subcutaneous (sternum) AT was collected from five Holstein-Friesian cows at a local abattoir. The tissue was rinsed in isopropanol for 60 s to minimize contamination and was then transported in sterile 50-mL tubes to the laboratory. All of the following steps were performed under sterile conditions and are based on the modified method of Grant *et al.* [59]. The outer layer of the AT was cut off, a block of approximately 3 g was cut into 1 mm^3^ pieces in cutting medium that contained Dulbecco’s modified Eagle’s medium low glucose (DMEM-LG; PAA, Pasching, Austria), 10 mg/mL penicillin/streptomycin (pen/strep) and 0.25 µg/mL amphotericin (all from PAA, Pasching, Austria). The cutting medium was drained off, and the AT pieces were transferred to 50-mL tubes and digested in DMEM-LG containing 2 mg/mL collagenase (244 U/mg) (Biochrom, Berlin, Germany) and 2% fatty acid-free BSA (Carl Roth, Karlsruhe, Germany). The samples were incubated at 37 °C for 15 min during which time the vials were mixed every 5 min. Each sample was then transferred to an incubator and further digested with shaking for 90 min at 37 °C, 370 rpm and at a 45° angle. The digested material was then sequentially filtered through 100-, 70- and 40-µm sterilized cell strainers into sterile 50-mL tubes and centrifuged at 800× *g* for 10 min at room temperature (RT). To eliminate erythrocyte contamination, 4 mL ultra pure sterile H_2_O was added, and the pellets were resuspended for 20 s. To adjust the osmotic pressure, the same amount of 2× PBS was added. After centrifugation at 800× *g* for 10 min at RT, the pellet was resuspended in growth medium (DMEM-LG, 10 mg/mL pen/strep, 0.25 µg/mL amphotericin, 33 µM biotin, 17 µM pantothenate, and 100 µM ascorbate (all substances were from AppliChem GmbH, Darmstadt, Germany, unless otherwise stated), supplemented with 10% fetal calf serum (FCS) (PAA) and seeded on 10 cm^2^ petri dishes. The medium was replaced after 24 h and on every 2nd day thereafter. After reaching confluence (90%–95%), the cells were washed twice with PBS, harvested with 0.5 g trypsin/EDTA and collected by centrifugation at 800 × *g* for 10 min at RT. The pellets were resuspended in freezing medium that contained Dulbecco’s modified Eagle’s medium high glucose (DMEM-HG) (PAA), 20% FCS and 10% dimethyl sulfoxide (DMSO, Carl Roth), frozen consecutively at −20 and −80 °C for 24 h each and then stored in liquid nitrogen until further use.

### 4.2. Differentiation of Bovine Preadipocytes

A pool of equal proportions of preadipocytes from five different animals was seeded in 25 cm^2^ flasks at a density of 2500 cells per cm^2^ and cultured with growth medium in a humidified atmosphere of 95% air and 5% CO_2_ at 37 °C for 24 h. To induce differentiation of the preadipocytes, 0.5 mM 3-isobutyl-methylxanthine (IBMX) (Applichem), 0.25 µM dexamethasone, 5 µg/mL bovine insulin (both from Sigma-Aldrich, St. Louis, MO, USA) and 5 µM troglitazone (Cayman Chemical, Ann Arbor, MI, USA) were added to DMEM-HG containing 5% FCS and 10 mg/mL pen/strep for 48 h. The cells were then maintained in post-differentiation medium (DMEM-HG, 5% FCS, 10 mg/mL pen/strep, 5 µg/mL bovine insulin and 5 µM troglitazone). The media were replaced every 2nd day, and the cells were used for the experiment at day 12 after the initiation of differentiation. Only adipocytes from those animals were used for the experiment that showed a differentiation rate of 60% at this time period, which was documented by the accumulation of lipid droplets (Oil Red O staining, 0.2%).

### 4.3. Treatment of the Cells with NA

Prior to the treatments at day 12, the cells were cultured in insulin-free DMEM-HG containing 5% FCS and 10 mg/mL pen/strep for 24 h and then serum starved in DMEM-LG supplemented with 0.1% BSA for 4 h. According to Tunaru *et al.* using CHO-K1 cells and Soliman *et al.* using differentiated bovine preadipocytes [7,60], the adipocytes were incubated for 16 h, with or without 100 ng/mL PTX (Sigma-Aldrich), to inhibit G_i_/G_o_-protein coupled signaling. The cells were then treated with 10 or 15 µM NA (Sigma-Aldrich) for 12 or 24 h, respectively. Equal volumes of PBS were applied in place of the PTX and NA for the controls. At the end of the incubation time, the supernatant was collected and stored at −20 °C until analysis. The adherent adipocytes were washed twice with ice-cold PBS, lysed with 1 mL Qiazol (Qiagen, Hilden, Germany) and subsequently frozen at −80 °C for total RNA and protein extractions. The differentiation and treatment procedure was independently repeated five times (*n* = 5).

### 4.4. RNA Extraction, cDNA Synthesis and mRNA Quantification

Total RNA from the Qiazol cell lysate (Qiagen, Hilden, Germany) was isolated with the Invitrap Universal RNA mini kit (Stratec Molecular, Berlin, Germany). To do this, the aqueous Qiazol phase was transferred and mixed with an equal volume of lysis solution TR containing DNA-binding particles. After binding of the residual DNA to the particles, the samples were centrifuged, and the supernatants containing the total RNA were subsequently purified with spin columns (Invitrap Universal RNA mini kit, Stratec Molecular). Total RNA concentrations and purities were analyzed by absorbance readings at 260 and 280 nm (Nanodrop 1000, peQLab Biotechnology, Erlangen, Germany). The total RNA integrity was verified using denaturing RNA gel electrophoresis. Additionally, the quality of the total RNA was rechecked in random samples by microcapillary electrophoresis using the Bioanalyzer 2100 and the RNA 6000 Nano Kit system (Agilent, Waldbronn, Germany) to determine the RNA integrity numbers (RIN = 9.05 ± 0.73). For cDNA synthesis, reverse transcription of 350 ng of total RNA per 20 µL reaction volume was performed with RevertAid™ reverse transcriptase (Thermo Fisher, Schwerte, Germany) according to the manufacturer’s instructions with the exception that only 1 µL of dNTP mix was used (10 mM of each dNTP, Thermo Fisher). Reverse transcription was performed in a Multicycler PTC 200 (MJ Research, Watertown, MA, USA) using a negative template control and one control per run in which no reverse transcriptase was included. For inter-run normalization of the PCR runs, the pooled RNA was additionally reverse transcribed. Reverse transcription was performed in duplicate for each sample, and the duplicate products were then combined for quantitative PCR (qPCR).

Characteristics of the primers and the quantitative real-time PCR conditions are displayed in Table 2. The selection of the reference genes and the data normalization were based on the methods of Saremi *et al.* [61] using qbase^+^ (Biogazelle, Gent, Belgium). Triplicates with 2 µL cDNA (diluted 1:4) as the templates and 5 µL SYBR Green Jump Start Taq Readymix (Sigma-Aldrich) or DyNAmo ColorFlash SYBR Green qPCR kit (Thermo Fisher) were run in total volumes of 10 µL in an Mx3000P cycler (Stratagene, Amsterdam, The Netherlands). A negative template control, a control lacking reverse transcriptase and an additional two inter-run calibrators were run in each run. The efficiencies were estimated with PCR amplicon standard curves. The PCR products were verified by sequencing.

**Table 2:** Characteristics of the primers and the quantitative real-time PCR conditions.

Gene ^a^	Forward Primer Sequence (5'-3') Reverse Primer Sequence (5'-3')	Acc. No.^d^	Base Pairs	Con. (nM) ^e^	Mean Cq ^f^	Annealing (s/°C) ^g^	Efficiency
**AdipoR1 ^b^**	GCTGAAGTGAGAGGAAGAGTC GAGGGAATGGAGTTTATTGCC	NM_001034055	118	800	23.9	35/61	99.9
**AdipoR2 ^b^**	GGCAACATCTGGACACATC CTGGAGACCCCTTCTGAG	NM_001040499	200	400	24.2	45/60	90.7
**GPR109A ^c^**	GGACAGCGGGCATCATCTC CCAGCGGAAGGCATCACAG	XR_028237	140	200	31.9	30/61	86.5
**FABP4 ^b^**	CATCTTGCTGAAAGCTGCAC AGCCACTTTCCTGGTAGCAA	X89244	160	800	22.9	30/60	120.4
**Chemerin ^b^**	GAAGAAAGACTGGAGGAAAG TTGAACCTGAGTCTGTATGG	FJ594406	139	200/100	23.2	60/60	89.1
**MARVELDI ^c^**	GGCCAGCTGTAAGATCATCACA TCTGATCACAGACAGAGCACCAT	NM_001101262	100	400	23.4	45/59	101.2
**EMD ^b^**	GCCCTCAGCTTCACTCTCAGA GAGGCGTTCCCGATCCTT	NM_203361	100	400	23.4	45/59	101.7
**LRP10 ^b^**	CCAGAGGATGAGGACGATGT ATAGGGTTGCTGTCCCTGTG	Bc149232	139	400	22.7	30/61	101.1
**EIF3K ^b^**	CCAGGCCCACCAAGAAGAA TTATACCTTCCAGGAGGTCCATGT	NM_001034489	125	400	23.4	45/59	97.5
**POLII ^b^**	GAAGGGGGAGAGACAAACTG GGGAGGAAGAAGAAAAAGGG	X63564	86	800	23.1	60/60	97.4

^a^ AdipoR1: adiponectin receptor 1 [62], AdipoR2: adiponectin receptor 2 [62], GPR109A: G-protein coupled receptor 109A [62], FABP4: fatty acid binding protein 4 [63], MARVELD1: marvel domain containing 1 [64], EMD: emerin [64], LRP10: lipoprotein receptor-related protein 10 [63], EIF3K: eukaryotic translation initiation factor 3, subunit K [64], POLII: RNA polymerase II [63]; ^b^ DyNAmo ColorFlash SYBR Green qPCR kit (Thermo Fisher); ^c^ SYBR Green Jump Start Taq Readymix (Sigma-Aldrich); ^d^ NCBI Accession Number; ^e^ Concentrations for each primer (forward/reverse); ^f^ median cycle threshold; ^g^ Initial denaturation for 10 min at 90 °C; denaturation for 30 s at 95 °C, extension at 72 °C, 60 s.

### 4.5. Protein Extraction from the Cell Lysate

For the analysis of the AMPK activation due to the NA treatment (15 µM NA for 24 h and the corresponding controls), DNA was precipitated from the Qiazol interphase and phenol phase. The resulting phenol-ethanol supernatant was used, after centrifugation, for protein precipitation according to the methods of Chey *et al.* with minor modifications [65]. Briefly, 1.75 mL of 100% ethanol was added to 700 µL of the phenol-ethanol supernatant, followed by 470 µL bromochloropropane (Applichem) and 1.4 mL H_2_O. After centrifugation (3900× *g*, 30 min, RT), the upper aqueous phase was discarded, and 1 mL 100% ethanol was added for protein precipitation. Pelleting of the protein was performed by centrifugation (3900× *g*, 10 min, RT); to purify the pellet, the addition of ethanol and centrifugation were repeated once. Next, the pellet was dried for 10 min and subsequently dissolved in 300 µL 4% SDS (Carl Roth) by shaking for 30 min at 55 °C. The protein content was quantified according to the method of Bradford [66] using the Nanodrop 1000 (Peqlab Biotechnology). The samples were frozen at −20 °C until the Western blot analyses.

### 4.6. Western Blot

To detect α-AMPK and pAMPK, 9 µg of total cell protein was treated with Laemmli buffer and reduced with 4% dithiothreitol (DTT) (Applichem), heated for 5 min at 95 °C, centrifuged for 5 min at 10,000× *g* at 4 °C, and subsequently loaded in duplicate on a 10% Mini-PROTEAN TGX Precast Gel (Bio Rad Laboratories, Munich, Germany). After electrophoresis, the fractionated proteins were transferred to a polyvinylidene difluoride (PVDF) membrane (GE Healthcare, Buckinghamshire, UK) using the Trans Turbo Blot (Bio Rad Laboratories). To minimize nonspecific binding, the membranes were incubated in tris-buffered saline containing 0.05% Tween 20 (TBST) and 10% Rotiblock (Carl Roth) for 60 min at RT. The membranes were cut horizontally at 50–55 kDa. The upper parts of the membranes were either incubated with the primary rabbit antiserum against α-AMPK or its phosphorylated form (pAMPK) (both from Cell Signaling, Danvers, MA, USA, and both 62 kDa) in dilutions of 1:500 or 1:200, respectively, in TBST with 5% BSA overnight at 4 °C. The bottom parts of the membranes with proteins ≤50 kDa were incubated with a primary mouse antibody against β-actin (42 kDa) (Biovision, Milpitas, CA, USA) diluted to 1:6000 in blocking solution under the same conditions. After rinsing, a horseradish peroxidase-labeled secondary goat anti-rabbit antibody (1:50,000; Cell Signaling) or a horseradish peroxidase-labeled secondary goat anti-mouse antibody (1:20,000, SouthernBiotech, Birmingham, ALA, UK) was applied for 60 min at RT. The immunocomplexes were revealed using the enhanced chemiluminescence detection system (GE Healthcare), and densitometric analyses were performed using the Versa Doc 1000 and the Image Lab software (both from Bio Rad Laboratories). The intensities of the specific bands were normalized to the β-actin values for the internal standards. To compare the band intensities from different membranes, a pooled sample of lysed 3T3-L1 differentiated adipocytes was electrophoresed and blotted in duplicate on each membrane for use as a reference standard. The mean intensities of the duplicate bands of the samples in relation to the means of the standards (100%) were calculated. Due to the missing values for pAMPK, the obtained optical densities of AMPK were matched against the reference standard.

### 4.7. Measurement of Adiponectin Secreted from Bovine Adipocytes

The adiponectin concentrations in the cell culture supernatants were quantified with a bovine adiponectin-specific ELISA that was developed in-house [67]. The intra- and interassay coefficients of variation were 7% and 11%, respectively.

### 4.8. Statistical Analyses

The data were analyzed using IBM SPSS 20 (IBM, Ehningen, Germany) and are presented as the means ± the SEMs. The results of the NA-free controls did not vary across time; thus, these data were merged across the PTX (+) and PTX (−) treatments for further analyses. For comparisons within the treatment groups and between the treatments and controls, ANOVAs with Bonferroni post-hoc analyses, depending on the homogeneities of the variances, was performed. Student’s *t*-tests were used to compare the PTX (+)- and PTX (−)-treated samples. Statistical significance was declared at *p* ≤ 0.05.

## 5. Conclusions

In conclusion, treatment with NA stimulated the secretion of adiponectin, the expression of AMPK protein, and the expression of chemerin mRNA in bovine adipocytes and therefore might improve insulin sensitivity and/or adipocyte metabolism in dairy cows. The inhibitory effect of PTX and the increase in the abundance of GPR109A mRNA suggest a G_i_/G_o_-protein-coupled receptor signaling pathway in cows and we speculate that GPR109A is at least partially involved in the NA-stimulated adiponectin and AMPK signaling pathways in bovine adipocytes.
